# Closed-loop protocol for inferior vena cava filter management significantly improves retrieval: a competing-risk analysis of a 10-year cohort

**DOI:** 10.1007/s11739-026-04361-7

**Published:** 2026-04-29

**Authors:** Víctor Manuel Martínez-Castilla, Elena Rodríguez-Llamas, Ana de-Miguel-Álvarez, Marina López-Rubio, Marta Olimpia Lago-Rodríguez, Rubén Alonso-Beato, Arturo Álvarez-Luque, Enrique Calleja-Cartón, Lucía Ordieres-Ortega, Sergio Moragón-Ledesma, Pablo Demelo-Rodríguez, Luis A. Alvarez-Sala-Walther, Francisco Galeano-Valle

**Affiliations:** 1 Venous Thromboembolism Unit. Internal Medicine Department. Hospital General, Universitario Gregorio Marañón, C/. Doctor Esquerdo, 46, 28007 Madrid, Spain; 2 School of Medicine, Universidad Complutense de Madrid, Madrid, Spain; 3 Sanitary Research Institute Gregorio Marañón, Madrid, Spain; 4 Division of Interventional Radiology Department of Radiology, Hospital General Universitario Gregorio Marañón, Madrid, Spain

**Keywords:** Anticoagulation, Bleeding risk, Deep venous thrombosis, Inferior vena cava filters, Pulmonary embolism

## Abstract

Inferior vena cava (IVC) filters are indicated only in acute venous thromboembolism (VTE) with an absolute contraindication to anticoagulation and should be retrieved once anticoagulation can be resumed. However, real-world retrieval rates remain low and highly variable. The objective is to assess whether implementing an institutional protocol improved appropriateness of IVC-filter placement and retrieval rates at 90 and 180 days, and to evaluate safety outcomes. Retrospective pre–post single-center study including adults who received an IVC filter between January 2015 and July 2025. Co-primary outcomes were appropriateness at placement (acute VTE ≤ 30 days plus absolute contraindication to anticoagulation) and retrieval, evaluated at 90 and 180 days using Fine–Gray competing-risk models. Among 234 patients (pre *n* = 182; post *n* = 52), cancer prevalence was similar (40.1% vs 38.5%). Appropriateness increased from 74.7% to 88.5% (risk ratio 1.18, 95% CI 1.04–1.35; *p* = 0.036). Retrieval improved at day 90 (41.2% → 65.4%, *p* = 0.020) and at day 180 (41.8% → 73.1%, *p* < 0.001). In Fine–Gray models, the post-protocol period was associated with a higher cumulative incidence of retrieval (adjusted sub-HR 1.68 [1.16–2.43] at 90 days; 1.92 [1.39–2.66] at 180 days), consistent with cause-specific Cox estimates. Procedure-related complications did not increase. Although overall mortality was unchanged, retrieval was independently associated with lower 180 days (adjusted HR 0.20 [0.08–0.48]). Implementing a closed-loop, guideline-aligned protocol improved appropriateness and substantially increased IVC-filter retrieval without compromising safety. Older age and active cancer remained barriers to retrieval, highlighting priority groups for targeted follow-up strategies.

**Graphical Abstract:**

**Figure:**
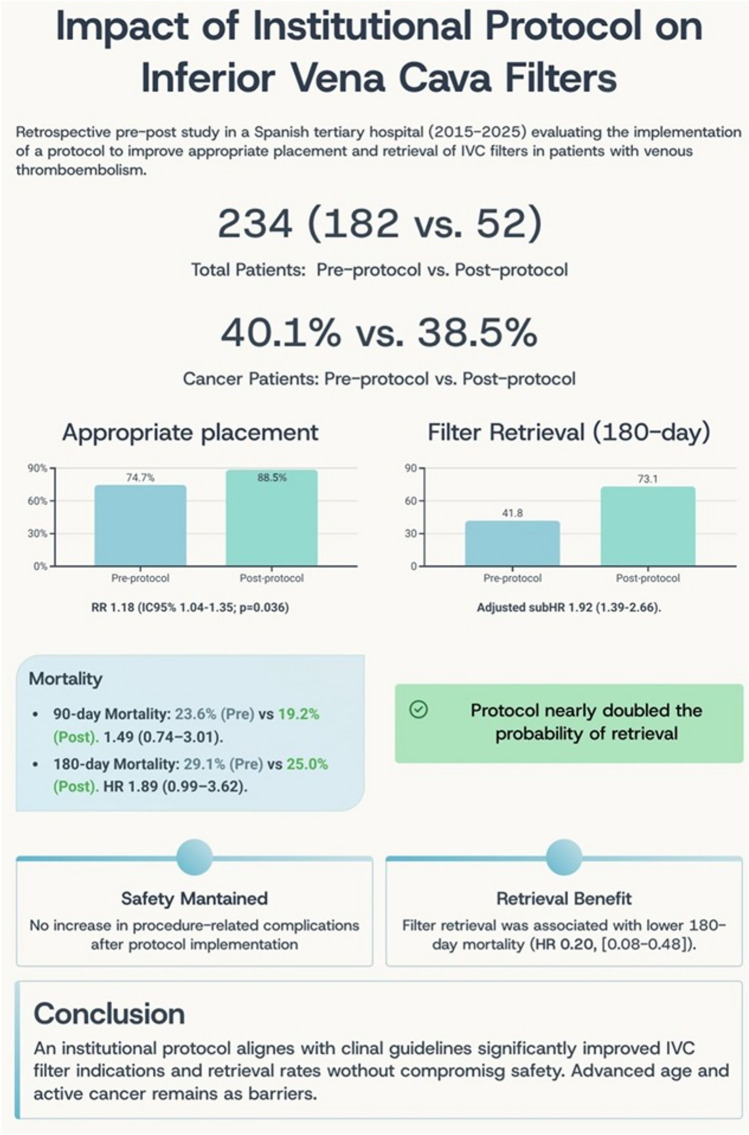

## Introduction

Venous thromboembolism (VTE), encompassing deep vein thrombosis (DVT) and pulmonary embolism (PE), is a major cause of morbidity and mortality. Inferior vena cava (IVC) filters were conceived to prevent embolization to the pulmonary circulation when anticoagulation is not feasible or has failed. Contemporary guidance converges on a restrictive strategy: filters should be reserved for patients with objectively confirmed acute VTE and an absolute contraindication to anticoagulation, and should be retrieved promptly once anticoagulation can be restarted [1–3].

Evidence syntheses underscore the narrow benefit–risk window. A 2017 meta-analysis [4] reported reduced subsequent PE with filters but increased risk of DVT and no mortality benefit. A 2018 meta-analysis [5], focused on retrievable devices failed to show reductions in PE recurrence or mortality. In anticoagulated patients, adding a retrievable filter does not improve survival and increases DVT risk [4], and Cochrane findings are concordant [5]. Filter-related complications (thrombosis/occlusion, penetration, migration, tilt, fracture) rise dwell time, reinforcing the need for early retrieval [6].

Despite consensus guidance, practice remains heterogeneous. Retrieval rates are often low and variable across centers, with only a minority of devices retrieved within recommended time frames [7]. Large-scale data from US Medicare patients illustrate this gap, showing a cumulative retrieval incidence of 15.3% at a median of 1.2 years [8]. Certain populations, particularly those with active cancer, face additional barriers to retrieval due to competing priorities and poorer prognosis [9]. Prolonged dwell, in turn, increases complication burden and retrieval complexity [6].

Quality-improvement (QI) strategies can address these shortfalls. Integrated electronic health record (EHR) protocols that assign ownership, automate reminders, and schedule checkpoints increase retrieval attempts and completions [10]. Multidisciplinary programs that formalize collaboration between interventional radiology, thrombosis services, and nursing coordination improve follow-up reliability and shorten dwell time [11]. Methodologically, before-and-after evaluations should account for death as a competing event; conventional survival methods that censor death can overestimate retrieval probabilities in frail cohorts [10, 12]. Competing-risk models (e.g., Fine–Gray) provide more realistic estimands, and adjustment for case-mix differences, particularly age and cancer, is essential.

Against this background, our VTE unit implemented, on April 1, 2023, an institutional protocol that (i) restricts IVC filter placement to acute VTE (≤ 30 days) with an absolute contraindication to full-dose anticoagulation; (ii) mandates early reinitiation of anticoagulation once safe; and (iii) embeds a closed-loop retrieval pathway with clear service ownership, scheduled appointments, EHR prompts, and pre-extraction safety checks. Additional information on the filter management protocol is provided in the Online Resource 1 [1–3, 10, 11].

We conducted a retrospective, single-center, quasi-experimental pre–post analysis spanning January 2015–July 2025 to assess the protocol’s impact on two co-primary quality domains: appropriateness of indication at placement and filter retrieval at 90 and 180 days. Recognizing the high competing risk of death in this population, we prespecified Fine–Gray competing-risk analyses for retrieval, alongside cause-specific Cox models, and adjusted for age and active cancer to address key confounders. This evaluation provides European single-center evidence on whether a guideline-aligned, system-level intervention can tighten indications and materially improve retrieval without compromising safety.

## Methods

### Study design and setting

Retrospective, single-center, quasi-experimental pre–post study in a tertiary hospital in Spain. We compared clinical practice before and after implementation of an institutional IVC filter protocol on April 1, 2023 [1–3, 10, 11].

### Study population

We included adults (≥ 18 years) who underwent IVC filter placement between January 1, 2015 and July 31, 2025, with a clinical follow-up available within our institution. Exclusion criteria were (i) Insufficient clinical information to adjudicate indication or outcomes and (ii) transfer to a non-affiliated center with loss to follow-up.

The pre-protocol period spanned January 2015–March 2023, and the post-protocol period April 2023–July 2025.

### Institutional protocol (intervention)

The protocol standardized three management steps:

- Indication: placement restricted to objectively confirmed acute VTE (≤ 30 days) plus absolute contraindication to therapeutic anticoagulation.
- Anticoagulation resumption: re-initiation as soon as clinically safe.
- Closed-loop retrieval: scheduled reassessment, fixed service ownership, electronic reminders, and structured pre-retrieval review [10, 11].

Additional information on the filter management protocol is provided in the Online Resource 1.

### Data collection

Patients were identified from the interventional radiology registry and cross-checked in the EHR. Trained investigators extracted demographics, comorbidities, VTE presentation, indication details, filter-related characteristics, anticoagulation management, complications, retrieval attempts, and clinical outcomes.

### Variables and definitions

Appropriate indication for filter placement required: (1) acute VTE (≤ 30 days) and 2) absolute contraindication to anticoagulation, including: active major bleeding, severe thrombocytopenia (< 50.000/mm^3^), recent neurosurgery, high-risk intracranial lesion, planned high-risk surgery, bleeding diathesis, recent ischemic stroke or traumatic brain injury. Filter retrieval was considered successful when complete retrieval was documented procedurally.

Newly diagnosed PE was defined as a filling defect observed in pulmonary artery CT or a ventilation/perfusion mismatch on lung scintigraphy. Deep venous thrombosis (DVT) recurrence was defined as thrombus in a new location from the original DVT, confirmed by duplex ultrasound imaging or CT. Major bleeding was defined according the ISTH definition as acute, clinically overt bleeding associated with one or more of the following: a decrease in hemoglobin of at least 2 g/dL, transfusion of 2 or more units of red blood cells, bleeding at a critical site (intracranial, intraspinal, intraocular, pericardial, intraarticular, intramuscular with compartment syndrome, or retroperitoneal), bleeding requiring surgical intervention, or fatal bleeding. Clinically significant non-major bleeding was defined as any bleeding that is not classified as major but requires medical intervention [13].

Data regarding study outcomes were independently collected by at least two investigators. A standardized data collection process was implemented to ensure consistency and accuracy across the study.

### Outcomes

Two co-primary outcomes were defined.

- Proportion of filters placed for appropriate indication.
- Filter retrieval at 90 and 180 days.

Secondary outcomes included time to retrieval; a composite outcome occurring, while the filter remained indwelling: including all-cause mortality, VTE recurrence, major bleeding, or any filter-related complication; 90-day mortality unrelated to VTE; 90-day major bleeding; 90-day and 180-day all-cause mortality.

### Follow-up

Patients were followed for at least 180 days after placement.

### Ethics and risks

This study was approved by the Institutional Ethics Committee (order FiltrosVC) and conducted in accordance with the Declaration of Helsinki and its subsequent revision in Fortaleza. Data were pseudonymized and stored on secure, access-restricted servers. Informed consent was waived due to the retrospective design.

### Statistical analysis

Qualitative variables were presented as frequencies and percentages. Quantitative variables were expressed as mean and standard deviation (SD) if normally distributed, and as median and interquartile range (25th–75th percentile) otherwise. Group comparisons used χ^2^ or Fisher’s exact test for categorical variables, and Student’s *t* test or Mann–Whitney *U* test for continuous variables. Spearman’s correlation was used for associations between quantitative variables. Risk ratios (RR) with 95% confidence intervals (CI) were reported for key proportions, and Hodges–Lehmann estimates were provided where informative.

Two co-primary quality outcomes were pre-specified: (1) appropriateness of indication at placement (binary, proportion per period) using χ^2^. (2) 90-day and 180-day IVC filter retrieval and as cumulative incidence over follow-up accounting for death as a competing event. Competing-risk analysis for time to filter retrieval was modeled used Fine–Gray subdistribution (event = retrieval; competing event = death), reporting subdistribution hazard ratios (sHR). Because death precludes the possibility of filter retrieval, it represents a competing event. Therefore, Fine–Gray models estimate the cumulative incidence of retrieval while appropriately accounting for the probability of death, providing a clinically more realistic estimate than standard survival methods that censor deaths. Cause-specific Cox models (death censored) were also fitted to estimate the instantaneous hazard of retrieval among patients still at risk. Both approaches were performed in bivariable and prespecified multivariable models adjusting for period, age, and active cancer.

Secondary outcomes: (1) The Mann–Whitney *U* test was used to compare the median duration of time to retrieval periods between groups; (2) Composite outcome (all-cause mortality, VTE recurrence, major bleeding, or any filter-related complication) occurring, while the filter remained indwelling using χ^2^. (3) Each individual outcome component was also evaluated separately. (4) Multivariable analysis for 90-day and 180-day all-cause mortality: exploratory Cox models adjusted for filter retrieval, period, age, active cancer, and CKD.

Statistical analyses were conducted using IBM SPSS Statistics v20 (SPSS Inc., Chicago, IL) and Epidat 3.1 (Xunta de Galicia, OPS, Epidat 3.1, Coruña, 2006, Washington, DC), using two-sided *α* = 0.05.

## Results

**Fig. 1:**
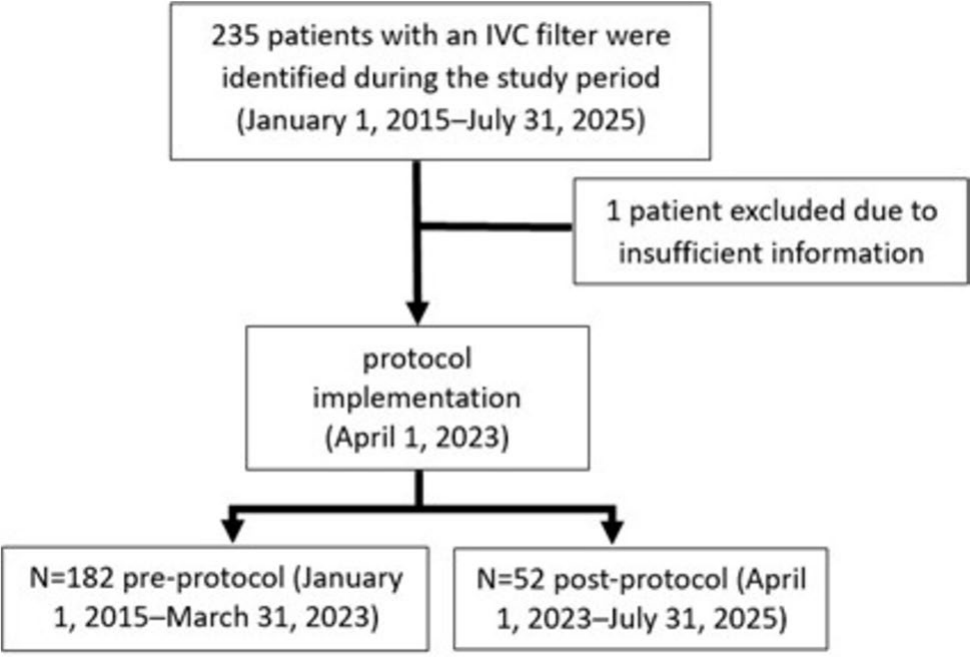
Flow-chart diagram

A total of 235 patients received an IVC filter during the study period; one was excluded due to insufficient data (Fig. 1). The final cohort included 234 patients (182 pre-protocol, 52 post-protocol). Baseline characteristics and VTE presentation are shown in Table 1; active cancer was present in 40.1% and 38.5% of patients, respectively.

**Table 1:** Baseline characteristics, VTE presentation and indications for IVC filters

Variable	Pre-protocol period (*n* = 182)	Post-protocol period (*n* = 52)	*p* value
Baseline characteristics			
Median age, years (IQR)	71 (56–80)	62.5 (53–76.7)	0.087
Sex male, % (*n*)	51.6 (94)	53.8 (28)	0.780
Arterial hypertension, % (*n*)	47.3 (86)	42.3 (22)	0.528
Chronic kidney disease, % (*n*) (eGFR < 60 mL/min/1.73 m^2^)	13.2 (24)	21.2 (11)	0.155
Hemoglobin < 12 g/dl, % (*n*)	66.5 (121)	65.4 (34)	0.883
Active cancer, % (*n*)	40.1 (73)	38.5 (20)	0.830
Gastrointestinal and Genitourinary	61.6 (45/73)	75 (15/20)	0.201
Brain metastasis	12.5 (9/73)	15 (3/20)	0.719
Central nervous system	9.7 (7/73)	5 (1/20)	0.681
Hematological	5.6 (4/73)	0 (0/20)	0.573
Other	10.9 (8/73)	5 (1/20)	0.43
VTE presentation			
PE, % (*n*)	69.4 (125/180)	70.6 (36/51)	0.875
PE location, % (*n*)			
Subsegmentary	8 (10/125)	11.1 (4/36)	0.517
Segmentary	44 (55/125)	27.8 (10/36)	0.08
Lobar	24.8 (31/125)	27.8 (10/36)	0.718
Principal	23.2 (29/125)	33.3 (12/36)	0.219
ESC stratification risk, % (*n*)			
Low risk	5.4 (7/125)	0 (0/36)	0.350
Intermediate–low risk	40 (50/125)	33.3 (12/36)	0.469
Intermediate–high risk	20.8 (26/125)	30.6 (11/36)	0.220
High risk	9.6 (12/125)	5.6 (2/36)	0.737
Incidental	24 (30/125)	30.6 (11/36)	0.427
DVT, % (*n*)	56.1 (101/180)	52.9 (27/51)	0.688
Proximal DVT (popliteal or proximal)	94.1 (95/101)	88.9 (24/27)	0.397
Distal DVT	5.9 (6/101)	11.1 (3/27)	0.397
Left side	49.5 (50/101)	51.9 (14/27)	0.973
Right side	45.5 (46/101)	25.9 (7/27)	0.066
Bilateral	5 (5/101)	22.2 (6/27)	0.011
No acute VTE at index*, % (*n*)	1.1 (2)	2 (1)	0.529
Contraindication to anticoagulation			
Absolute contraindication to anticoagulation, % (*n*)	74.7 (136)	88.5 (46)	0.036
Intracranial lesion with high hemorrhagic risk	8.8 (12/136)	8.7 (4/46)	1
Active major bleeding	80.9 (110/136)	76.1 (35/46)	0.485
Severe thrombocytopenia (platelet count < 50,000/mm^3^)	2.2 (3/136)	6.5 (3/46)	0.170
Recent intracranial or spinal surgery	4.4 (6/136)	19.6 (9/46)	0.003
Need for surgery during anticoagulant treatment	25.7 (35/136)	23.9 (11/46)	0.806
Hemorrhagic diathesis	1.5 (2/136)**	4.3 (2/46)**	0.265
Recent ischemic stroke	4.4 (6/136)	6.5 (3/46)	0.694
Recent traumatic brain injury	8.1 (11/136)	8.7 (4/46)	1
Relative contraindication to anticoagulation, % (*n*)	7.7 (14)	0 (0)	0.044
Clinically significant non-major bleeding	64.3 (9/14)	0 (0)	–
Previous bleeding non active at the moment of diagnosis of VTE	21.4 (3/14)	0 (0)	–
Non-severe thrombocytopenia (platelet count > 50,000/mm^3^)	14.3 (2/14)	0 (0)	–
Other indications for filter placement, % (*n*)	32.7 (36)	11.5 (6)	0.172
Primary VTE prophylaxis in a patient scheduled for general surgery	0.5 (1)	0 (0)	–
Primary VTE prophylaxis in a patient scheduled for orthopedic surgery	0.5 (1)	0 (0)	–
History of VTE 1.5–3 months prior and current contraindication to anticoagulation	11 (20)	5.8 (3)	0.406
History of VTE > 3 months prior and current contraindication to anticoagulation	1.1 (2)	0 (0)	–
Recurrence of VTE despite anticoagulation	3.3 (6)	1.9 (1)	1
Others	3.3 (6)	3.8 (2)	1

^*^ Defined as placements without objectively confirmed acute VTE ≤ 30 days at index

^**^Two cases of disseminated intravascular coagulation and two cases of liver dysfunction

*ESC* European Society of Cardiology, *DVT* deep venous thrombosis, *IQR*: interquartile range, *IVC* inferior vena cava, *PE* pulmonary embolism, *VTE*: venous thromboembolism

### Co-primary outcomes

Appropriate indications, defined as acute VTE with an absolute contraindication to anticoagulation, increased after protocol implementation, from 74.7% to 88.5% (*p* = 0.036; RR 1.18, 95% CI 1.04–1.35) (Table 1).

Filter-related characteristics and management appear in Table 2. Retrieval improved significantly at both day 90 (41.2% vs 65.4%, *p* = 0.020) and day 180 (41.8% vs 73.1%, *p* < 0.001). Among retrieved filters, dwell time was slightly longer post-protocol (median 34.5 vs 42 days, *p* = 0.023). Filters were generally retrieved under therapeutic-dose anticoagulation, although therapeutic dosing at retrieval was less frequent post-protocol (58.6% vs 46.2%, *p* = 0.016).

**Table 2:** Anticoagulation treatment, IVC filter placement, removal characteristics, 90-day and 180-day outcomes in patients with IVC filter placement

Variable	Pre-protocol period (*n* = 182)	Post-protocol period (*n* = 52)	*p* value
Filter characteristics			
Type of filter (model), % (*n*)			
Optease™	12.1 (22)	1.9 (1)	0.300
Celect Platinum IVC Filter™	87.9 (160)	98.1 (51)	0.300
Access route for filter placement, % (*n*)			
Femoral	86.8 (158)	84.6 (44)	0.684
Jugular	13.2 (24)	15.4 (8)	0.684
Right side	88.5 (161)	88.5 (46)	0.791
Left side	11.5 (21)	11.5 (6)	1
Specialty that indicated IVC filter placement			
Internal Medicine	61.5 (112)	69.2 (36)	0.310
Oncology	7.1 (13)	3.8 (2)	0.531
Intensive Care	11.7 (12)	5.8 (3)	1
Hematology	7.8 (9)	1.9 (1)	0.465
Neurosurgery	3.3 (6)	0 (0)	0.343
Emergency Department	2.7 (5)	0 (0)	0.589
General Surgery	2.7 (5)	3.8 (2)	0.653
Gastroenterology	6.2 (5)	5.8 (3)	0.381
Pulmonology	2.2 (4)	0 (0)	0.578
Others	4.4 (8)	7.7 (4)	0.456
Filter removal rate, % (n)	42.9 (78)	75 (39)	< 0.001
Filter removal rate at day 90, % (n)	41.2 (75)	65.4 (34)	0.02
Filter removal rate at day 180, % (n)	41.8 (76)	73.1 (38)	< 0.001
Duration of filter placement in patients with filter removed, days (median, IQR)	34.5 (17.5;50.25)	42 (24;72)	0.023
Anticoagulation resumed, % (*n*)	73.1 (133)	75 (39)	0.782
Anticoagulation dose at which filter was removed, % (*n*)			
Prophylactic dose	15.5 (9/58)	20.5 (8/39)	0.194
Intermediate dose	25.9 (15/58)	33.3 (13/39)	0.092
Therapeutic dose	58.6 (34/58)	46.2 (18/39)	0.016
Removal route for filter retrieval, % (*n*)			
Femoral	14.1 (11/78)	15.4 (6/39)	0.853
Jugular	85.9 (67/78)	84.6 (33/39)	0.853
IVC filter placement complications			
Caval wall perforation, % (*n*)	0.8 (1)	0 (0)	1
Involvement of adjacent organs, % (*n*)	1 (1)	0 (0)	–
Asymptomatic	0 (0)	0 (0)	–
Complications while carrying the filter or during removal			
Time since filter placement and the imaging test, days (median, IQR)	36.5 (17.5;69)	40 (16;76)	0.846
Imaging performed prior to filter removal, % (*n*)	44.5 (81)	51.9 (27)	0.344
CT	86.4 (70/81)	92.6 (25/27)	0.393
Venography	13.6 (11/81)	7.4 (2/27)	0.393
None	55.5 (101)	48.1 (25)	0.344
Pathological imaging findings, % (*n*)	24.7 (20/81)	25.9 (7/27)	0.458
Thrombosis below the filter	11.5 (21/182)	15.4 (8/52)	0.085
PE in a patient with a filter	1.6 (3)	0.9 (1)	1
Patients with > 1 attempt, % (*n*)	1.6 (3)	0 (0)	–
Need for surgical removal, % (*n*)	0 (0)	0 (0)	–
Need for stent placement, % (*n*)	0 (0)	0 (0)	–
Death-related complication, % (*n*)	0.5 (1)	0 (0)	–
90-day outcomes			
Mortality, % (*n*)	23.6 (43)	19.2 (10)	0.504
Cause of death, % (*n*):			
PE	7 (3/43)	10 (1/10)	1
Major bleeding	18.6 (8/43)	20 (2/10)	1
Infection	18.6 (8/43)	50 (5/10)	0.052
Cancer	25.6 (11/43)	20 (2/10)	1
Others	30.2 (13/43)	0 (0/10)	0.096
VTE recurrence, % (*n*)	6 (11)	1.9 (1)	0.473
PE	27.3 (3/11)	100 (1/1)	0.333
DVT	54.5 (6/11)	0 (0/1)	1
Major bleeding, % (*n*)	11 (20)	31 (9)	0.223
180-day outcomes			
Mortality, % (*n*)	29.1 (53)	25 (13)	0.560
Cause of death, % (*n*):			
PE	5.7 (3/53)	7.7 (1/13)	1
Major bleeding	17 (9/53)	15.4 (2/13)	1
Infection	17 (9/53)	46.2 (6/13)	0.058
Cancer	28.3 (15/53)	30.8 (4/13)	1
Others	32.1 (17/53)	0 (0/13)	0.015
VTE recurrence, % (*n*)	6.6 (12)	1.9 (1)	0.307
PE	25 (3/12)	100 (1/1)	0.308
DVT	58.3 (7/12)	0 (0/1)	0.462
Major bleeding, % (*n*)	12.6 (23)	19.2 (10)	0.228

*CT* Computed tomography, *DVT* deep venous thrombosis, *IVC* inferior vena cava, *IQR* interquartile range, *PE* pulmonary embolism, *VTE* venous thromboembolism

### Composite outcome, while the filter remained indwelling

The composite of all-cause mortality, VTE recurrence, major bleeding, or any filter-related complication during the period the filter remained indwelling occurred in 58.2% of pre-protocol patients vs 46.2% post-protocol (*p* = 0.12). Rates of individual events are detailed in Table 2. Procedure-related complications did not increase in the post-protocol period.

### All-cause mortality at 90 and 180 days

Crude mortality was similar between periods. At 90 days, mortality was 23.6% pre-protocol vs 19.2% post-protocol (*p* = 0.50); at 180 days, 29.1% vs 25.0% (*p* = 0.56). Most 90-day deaths in both periods were unrelated to VTE or major bleeding (73.3% and 70%, respectively).

### Device and procedural details

Most implants were retrievable filters. Femoral access was used for placement in the majority of cases (86.8% and 84.6%), while internal jugular access predominated for retrieval (85.9% and 84.6%). Anticoagulation was reinitiated before retrieval in 73.1% and 75% of cases. Standard retrieval techniques sufficed in nearly all patients, with few repeat attempts (1.6% and 0%). No retrieval-related deaths occurred, and procedural complications remained low (Tables 2 and 3).

**Table 3:** Multivariable analysis for filter retrieval at 90 and 180 days using both Cox proportional hazards and Fine–Gray competing risks regression models

Variable	HR (95% CI) (Cox regression)	*p* value	subHR (95% CI) (Fine–Gray)	*p* value
Filter retrieval at 90 days				
Post-protocol period	1.70 (1.13–2.56)	0.0116	1.68 (1.16–2.43)	0.006
Age (per year)	0.98 (0.97–0.99)	0.0023	0.987 (0.978–0.997)	0.011
Active cancer	0.44 (0.29–0.67)	< 0.001	0.591 (0.395–0.883)	0.010
Mortality	–	–	0.169 (0.073–0.392)	< 0.001
Filter retrieval at 180 days				
Post-protocol period	1.96 (1.32–2.91)	< 0.001	1.92 (1.39–2.66)	< 0.001
Age (per year)	0.98 (0.97–0.99)	0.0032	0.988 (0.979–0.997)	0.012
Active cancer	0.41 (0.27–0.63)	< 0.001	0.601 (0.399–0.903)	0.014
Mortality	–	–	0.237 (0.122–0.459)	< 0.001

The Cox model estimated the hazard ratio (HR) for filter retrieval, adjusting for post-protocol period, age, and active cancer

The Fine–Gray model estimated the subdistribution hazard ratio (subHR) for filter retrieval, additionally accounting for death as a competing event

### Multivariable analysis

In Cox models adjusting for period, age, and active cancer, the post-protocol period was associated with higher retrieval rates at both 90 days (hazard ratio [HR] 1.70, 95% CI 1.13–2.56; *p* = 0.012) and 180 days (HR 1.96, 95% CI 1.32–2.91; *p* =  < 0.001 (Table 3).

Using Fine–Gray competing risk models, the protocol effect persisted: at 90 days (subdistribution hazard ratio, sHR 1.78, 95% CI 1.22–2.61; *p* = 0.003) and at 180 days (sHR 2.01, 95% CI 1.41–2.87; *p* =  < 0.001).

**Fig. 2:**
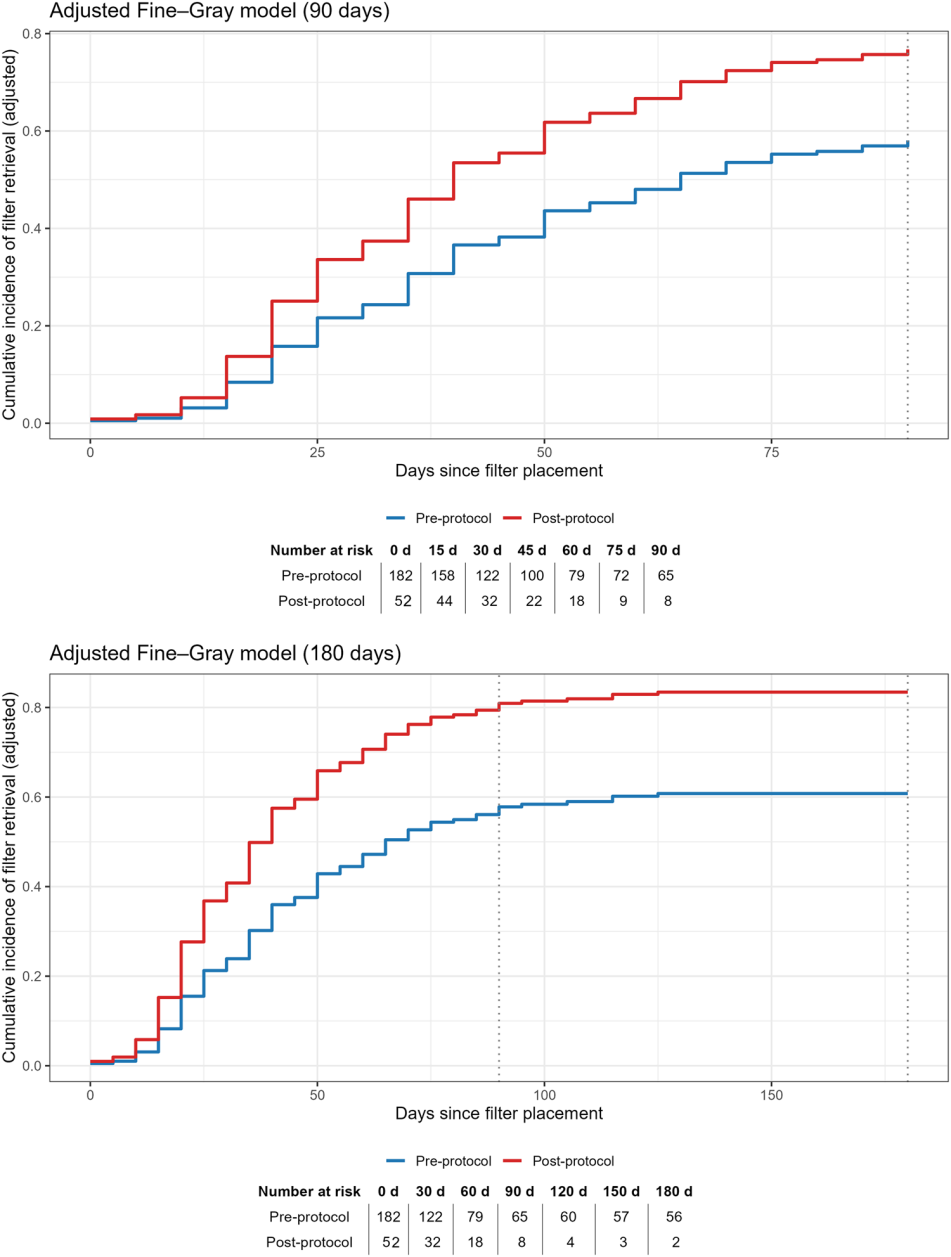
A, B Cumulative incidence of IVC filter retrieval to days 90 and 180 (Fine–Gray with death as the competing event; adjusted for period, age, and active cancer)

Using multivariable Fine–Gray competing risk models—adjusted for age and active cancer—the protocol effect persisted: at 90 days (sHR 1.68, 95% CI 1.16–2.43; *p* = 0.006) and at 180 days (sHR 1.92, 95% CI 1.39–2.66; p < 0.001) (Table 3). Older age and active cancer were associated with significantly lower probability of retrieval, and death markedly reduced the subdistribution hazard. Cumulative incidence curves diverged early and remained clearly separated (Fig. 2).

In Cox models for all-cause mortality (Table 4), active cancer and CKD independently predicted higher mortality. The post-protocol period showed a non-significant trend toward lower at 180 days. Filter retrieval remained strongly associated with reduced mortality at 90 days (HR 0.10, 95% CI 0.03–0.34; *p*= < 0.001) and 180 days (HR 0.20, 95% CI 0.08–0.48; *p* = < 0.001).

**Table 4:** Multivariable Cox proportional hazards regression model for all-cause mortality at 90 and 180 days, adjusted for filter retrieval, post-protocol period, age at filter placement, active cancer, and chronic kidney disease (CKD)

Variable	HR (95% CI)	*p* value	HR (95% CI)	*p* value
	Mortality at 90 days		Mortality at 180 days	
Filter retrieved	0.10 (0.03–0.34)	< 0.001	0.20 (0.08–0.48)	< 0.001
Post-protocol period	1.49 (0.74–3.01)	0.265	1.89 (0.99–3.62)	0.055
Age (per year)	1.00 (0.98–1.02)	0.937	1.00 (0.98–1.02)	0.943
Active cancer	1.86 (1.03–3.34)	0.040	2.04 (1.18–3.53)	0.011
Chronic kidney disease	1.95 (1.05–3.63)	0.034	1.75 (0.97–3.14)	0.062

*HR* hazard ratio

## Discussion

Implementing a closed-loop, guideline-aligned protocol [1–3] led to clear improvements in two domains central to high-quality IVC filter practice: stricter appropriateness at placement and markedly higher retrieval rates at clinically relevant timepoints. These benefits were consistent across estimands—both Fine–Gray cumulative incidence models and cause-specific Cox analyses—and remained robust after adjustment for age and active cancer. Importantly, this improvement occurred without an increase in procedure-related complications, and retrieval was strongly associated with lower short-term mortality.

These findings show that a pragmatic, system-level intervention can address the two main drivers of preventable harm in filter management: inappropriate placement and prolonged dwell time. The slightly longer dwell time among retrieved patients in the post-protocol period should be interpreted cautiously. Although the protocol incorporated structured follow-up and electronic reminders intended to facilitate timely retrieval, variability in clinical decision-making regarding the optimal timing of retrieval, scheduling logistics, or the relatively small number of retrieved filters in the post-protocol group may have contributed to this finding. Importantly, despite this difference, the protocol was associated with a substantial increase in the probability of filter retrieval at both 90 and 180 days. The lower proportion of patients receiving therapeutic-dose anticoagulation at the time of retrieval in the post-protocol period may reflect earlier reassessment of filter necessity under the structured protocol. In some cases, filters may have been removed once the contraindication to anticoagulation had sufficiently improved, even before full therapeutic anticoagulation was established. This strategy may facilitate timely filter retrieval while potentially minimizing bleeding risk during the transition back to therapeutic anticoagulation.

Our results align with contemporary guideline recommendations, which restrict filters to acute VTE with an absolute contraindication to anticoagulation and advocate for early retrieval once anticoagulation becomes feasible [1–3, 14]. Evidence from randomized trials and meta-analysis [15, 16] consistently shows no mortality benefit and an increased DVT risk when filters are added to appropriately anticoagulated patients, reinforcing the need for disciplined patient selection [9, 10]. Likewise, extensive literature shows that device-related complications rise with prolonged dwell time, supporting timely extraction [6, 17, 18].

The magnitude of improvement we observed is comparable to outcomes from structured quality-improvement programs and EHR-embedded pathways that assign clear ownership and incorporate automated reminders [10, 12, 14]. These programs have been proposed partly in response to persistently low retrieval rates in routine practice, with large datasets reporting retrieval in only a minority of patients. Indeed, recent data from 270,866 US Medicare beneficiaries showed that only 15.3% of filters were retrieved at a median follow-up of 1.2 years [7, 8]. Against this backdrop, the performance achieved after protocol implementation in our institution is encouraging and suggests that systematic, accountable pathways can meaningfully narrow the gap between evidence and practice.

A key methodologic contribution is the use of competing‐risk analyses of filter retrieval. Because death precludes retrieval, standard survival methods that censor death (e.g., Kaplan–Meier or standard Cox) can overestimate retrieval in frail populations. Reporting cumulative incidence functions (CIF) using Fine–Gray methods, therefore, provides a more realistic measure of program performance [8, 19]. Presenting both Fine–Gray and cause-specific Cox models also strengthens interpretability by capturing complementary aspects of the retrieval process [8, 19]. The consistency of the results across both modeling approaches further supports the robustness of the observed association between protocol implementation and improved filter retrieval.

Age and active cancer—both associated with poorer outcomes in prior studies—remained independent barriers to retrieval [9, 19–22]. These findings underscore the need for tailored strategies in these groups, such as scheduling retrieval at the time of anticoagulation resumption, incorporating filter status into oncology visits, or employing navigator-based follow-up [23, 24]. Similar targeted approaches have proven effective in trauma and multidisciplinary programs. Despite these challenges, protocol implementation still improved retrieval among these higher risk subgroups [24–26]. Nevertheless, the persistently lower retrieval rates observed in elderly patients and those with active cancer suggest that additional targeted interventions may be required, such as dedicated care pathways or closer multidisciplinary coordination between thrombosis, oncology, and interventional radiology teams.

Procedure-related complications did not increase after protocol implementation, and adverse events, while the filter remained indwelling decreased numerically. However, the study was not powered to detect rare adverse events, and therefore, conclusions regarding the safety of the intervention should be interpreted cautiously. This pattern is consistent with evidence that shorter dwell times reduce device-related risk [12–14].

The strong association between retrieval and lower short-term mortality should be interpreted cautiously. In observational settings, successful retrieval likely reflects clinical stabilization, recovery from the acute thromboembolic event, and the resumption of anticoagulation rather than a direct causal effect of filter retrieval itself. Patients eligible for retrieval are, therefore, likely to have a more favorable clinical trajectory, which introduces the possibility of confounding by indication and selection bias. Retrieval may thus act primarily as a marker of favorable prognosis and coordinated, guideline-driven care, although a potential contribution of reduced device-related risk cannot be entirely excluded.

This work has several strengths. These include the use of protocolized definitions aligned with international guidance [1–3, 16], near-complete follow-up, and robust statistical methodology incorporating prespecified competing-risk analyses to account for death as a competing event for retrieval.

However, several limitations should be acknowledged. The retrospective single-center design may limit the generalizability of the findings to other healthcare systems, institutional structures, or clinical practices, particularly outside Europe. The asymmetry in the number of patients between the pre- and post-protocol periods may also reflect changes in clinical practice after protocol implementation, particularly the restriction of indications for filter placement, and could introduce selection bias when comparing outcomes between periods.

In addition, the bundled nature of the intervention prevents identification of which specific protocol components—restriction of indications, early resumption of anticoagulation, or the closed-loop retrieval pathway—were most influential in improving outcomes. The observed improvements, therefore, reflect the effect of the protocol as a system-level intervention rather than the isolated impact of individual measures.

The long pre-intervention period and shorter post-intervention period also introduce the possibility of secular trends, such as gradual improvements in clinical awareness, operator experience, or institutional practices over time. Residual confounding, therefore, remains possible, as unmeasured factors such as clinician adherence to guidelines, institutional workflow changes, or variations in follow-up practices may have contributed to the observed results.

Finally, the study was not powered to perform detailed subgroup analyses according to specific types of anticoagulation contraindications. Future studies with larger cohorts may help clarify whether the impact of protocolized filter management varies across different clinical indications.

Future multicenter evaluations across diverse healthcare environments may help determine the broader applicability of this approach and identify which elements drive the greatest incremental gains.

## Conclusions

Institutional implementation of a guideline-aligned protocol significantly improved the appropriateness of IVC filter placement and markedly increased retrieval rates at 90 and 180 days, without increasing complications. However, a proportion of filters continued to be placed for indications not fully aligned with the protocol, highlighting the need for continued quality-improvement efforts. Older age and active cancer remained major barriers to retrieval. These data support protocolized, closed-loop pathways as an effective strategy to optimize filter management in routine practice.

## Data Availability

The datasets generated and/or analyzed during the current study are available from the corresponding author on reasonable request.
